# Sustained Retention, Viral Load Suppression and their Determinants Among Clients on HAART Enrolled Under Differentiated Service Delivery Models in Eastern Uganda

**DOI:** 10.21203/rs.3.rs-3377046/v1

**Published:** 2023-10-04

**Authors:** Jemba Brian, Sinani Waiswa, Joseph Balinaine, Rosaria Lomuria, Gift Gloria Nabutanyi, Emmanuel Ongala, Benjamin Opus, Mary Abwola Olwedo, Jacob Stanley Iramiot, Paul Oboth, Rebecca Nekaka

**Affiliations:** Busitema University; Busitema University; Busitema University; Busitema University; Busitema University; Busitema University; Busitema University; Soroti University; Busitema University; Busitema University; Busitema University

**Keywords:** Differentiated Service Delivery models, HIV/AIDS, Viral load suppression, Retention in care, Eastern Uganda

## Abstract

**Background;:**

Although Uganda rolled out Differentiated Service Delivery(DSD) models in June 2017 to improve retention and viral load suppression rates among clients on Highly Active Antiretroviral Therapy (HAART), these have remained low relative to the Joint United Nations Programme on HIV/AIDS(UNAIDs) targets of achieving 95% population with HIV tested, 95% of tested positive clients for HIV to be on Highly active Antiretroviral therapy and 95% of clients On Antiretroviral therapy be suppressing by 2030(95–95-95 UNAIDS targets). The purpose of this study was to determine sustained retention, viral load suppression and their determinants among clients on HAART enrolled under different Differentiated service delivery models in Katakwi district in Eastern Uganda.

**Methods;:**

A retrospective cohort study of clients enrolled on HAART in the different approaches of DSD who were active by 2017 and followed up to 2020 was done. The primary outcomes included sustained retention, viral load suppression and their determinants among clients HAART in different DSD approaches. Eight health facilities providing HAART services were purposively sampled and 771 clients on HAART were sampled out by simple random selection from a total population of 4742 clients on HAART in Katakwi district. We analysed retention, viral load suppression rates, and their determinants by logistic regression method using STATA.

**Results;:**

A total of 771 participants were sampled of whom 42.7% were male and 57.3% were female, with the mean age being 40 years. Retention rates at 95% CI of participants were 99.35% at 12 months, 94.03 at 24 months, 89.88% at 36 months and 84.57% at 48 months. The viral load suppression rates were 57.3% at 12 months, 70.3% at 24 months, 70.3% at 36 months and 69% at 48 months. Retention was higher in the community based DSD model as compared to the facility-based model. Viral load suppression was higher in the community based DSD models in which Community Drug Distribution Points had the highest achievement (92%) followed by Community Client-Led ART Distribution (79%) compared to the facility based DSD models in which Facility Based Individual Management performance (34.3%) was far below the set standard of 95%, followed by Facility Based Groups (65%) with Fast Track Drug Refill having relatively better performance (80.9%). Being 40–59 years, receiving care from the general hospital, being married, having good current adherence, being on the first line of the current regime and being a female are other predictors of viral load suppression, whereas being 40–59 years of age, having good current adherence, being on the current first-line regime and having no co-morbidities were predictors of good retention.

**Conclusions;:**

generally, facility and community based DSD models have demonstrated improved retention and viral load suppression. However, community-based models have shown to be more effective than facility-based models through mitigation of barriers to effective HIV/AIDS care of clients on HAART. Viral load suppression remained below the UNAIDs target of 95% by 2030, albeit it improved over time.

## Background

At the end of 2019, 38 million people of the world’s population were living with the Human Immune Deficiency Virus (HIV), HIV-related deaths contributed 690,000 deaths to general mortality and US $ 262 billion of the global economy was spent on HIV response in 2020. Approximately 59.7% of the HIV-related deaths occurred in low and middle income countries of which Uganda is inclusive (1). Approximately 1.5 million people in Uganda were living with HIV by 2019 with a funding gap of US $ 918 million estimated for the financial year 2019/2020 (2).

To end the HIV pandemic, the United Nations general assembly through the Joint United Nations Programme on Human Immune Deficiency Virus (HIV) / Acquired Immune Deficiency Syndrome (AIDS) committed to ending the HIV epidemic by 2030 through the famous 95–95-95 targets. The target was aimed at ensuring that 95% of all people living with HIV know their HIV status, of which 95% should receive sustained Highly Active Anti-Retroviral Therapy (HAART), and 95% of all people receiving HAART show viral load suppression by 2030 (3).

The last 95% UNAIDS target of achieving 95% viral load suppression is one of the goals of HAART with good retention as its strong predictor and crucial determinant of successful HIV treatment outcomes, though infrequently evaluated (4).

Sustained retention and hence viral load suppression by 95% of all clients on ART by 2030 could not be achieved with the pre-existing approaches of *“one coat fits all”* but rather improve their efficiency through approaches based upon the idea that HIV care delivery should be offered in different formats to suit or fit the varying needs of the clients (5)(6). As a result, many countries adopted several approaches such as Standardised Paediatrics Expedited Encounters for Drugs (SPEEDI) in Tanzania (7),Community ART distribution points (CAD), Community ART Groups (CAGs) plus 3months refills in Lesotho, Democratic Republic of Congo, Zimbabwe and Malawi. (8),(9),(10) and (5). They all showed positive outcomes in terms of retention of HAART clients and Viral load suppression.

Uganda joined other countries that subscribe to the United Nations and adopted innovative and efficient strategies to delivering care and treatment strategies and address the needs of different sub-populations of clients under HIV care through various Differentiated Service Delivery (DSD) models. Differentiated Service Delivery models to Human Immunodeficiency Virus (HIV) treatment and care refers to the strategic mix of approaches to address the specific requirements of the sub-groups of clients living with HIV. This is contrary to the previous approach of *one coat fits all* to HAART delivery where every person was treated the same way. The objective of DSD is to address individual needs, inform targeted interventions with better outcomes among clients, improve coverage and quality of care (11). The DSD models adopted by the Ministry of health Uganda include: Facility Based Individual Management (FBIM) for clients needing extra attention e.g. newly initiating HAART clients; Facility Based Group (FBG) for complex or stable clients in need of peer support, e.g. adolescent groups; Fast Track Drug Refill (FTDR) involving pick up from dispensing points or pharmacy after going via the triage desk; Community Client Led ART Delivery (CCLADs) in which clients form groups whose members (3–6) rotate in drug pick-ups; Community Drug Distribution Points (CDDPs) which involve clients (groups of about 50) picking up drugs and receiving their clinical evaluation when due from a community outreach point (11).

Uganda’s viral load suppression rate was estimated to be at 87% (12) and lower for Katakwi district about 85.7% in 2019 (District Health Information System 2(DHIS-2)). This could be due to low retention rates in Katakwi district noticed at about 59%(DHIS-2) despite the efforts by the Ministry of Health through different DSD approaches, which hinders Uganda as a country from achieving the 95% viral load suppression of ART clients. The purpose of this study was to determine sustained retention, viral load suppression and their determinants among clients receiving HAART in Katakwi District in Eastern Uganda.

## Methods and Materials

### Study Design

The study design used was a retrospective cohort study among people living with HIV receiving HAART from the eight ART accredited health facilities in Katakwi district enrolled on HAART by 2017 before being allocated DSD models. The Originally allocated DSD models were used as the cohorts and each individual ART client was followed up to 2020 and the outcome as viral load suppression and retention assessed in 2020 and compared with the baseline of 2017. ART clinics assigned clients approaches of DSD models depending on categories as either being stable or unstable. Stable clients are people living with HIV on current ART regimen for more than 12 months, virally suppressed in last 12 months, WHO clinical stage 1 or 2, TB clients who have completed 2 months intensive phase treatment, on 1st or 2nd line regimen and demonstrated good adherence in the last 6 consecutive months or else unstable. The clients are allocated to facility based individual management (FBIM) are unstable clients who need extra attention like newly initiating, sick needing multi-disease management, or virally non-suppressing clients from other models. The clients allocated to facility-based groups (FBGs) are either stable or unstable desiring peer support. Clients allocated to fast-track drug refills (FTDR) are stable but desire to pick their refills from picking points and pharmacy. Clients are allocated to the community client-led ART delivery (CCLAD) if they are stable and come from same locality and are able to form small groups. Clients are allocated to community drug distribution points are stable and live in an isolated community and consent to being part of a group bigger than CCLAD in which they pick their refills from a community outreach.

### Study Area

The study was conducted in Katakwi District which is located in the North-Eastern region of Uganda, lying between longitudes 33° 48’ E − 34° 18’ E and latitudes 1° 38’ N – 2° 20’ N.

It shares borders with the Districts of Napak in the North, Nakapiripirit in the East, Amuria in the West & North-West, and Soroti in the South –West, Kumi, and Ngora in the South.

The District Headquarters are situated in Katakwi Town Council, a road distance of about 380 km from Kampala (National capital city) by the most direct route and lies approximately between 1,050 − 1,130 m above sea level. The district has a total area of 2,507 sq. km., land areas is 2,177 sq.km and open water area and swamps is 177 sq. km. Land under cultivation is 720 sq. km, land under forest is 98.2 sq. km. and others are 53.5 sq. km. The district landscape is generally a plateau with a gently undulating plain with hills and Inselbergs.

### Health Services

The Katakwi District Health system / department is comprised of one District Hospital and one health sub-District, Usuk Health Sub-District. It is manned and coordinated by the District Health Office headed by the District Health Officer. The department is mandated with the delivery of medical and health services which include: managing and functionalizing all health centres, providing maternity and child healthcare services, vector control, controlling communicable diseases, especially malaria, HIV/AIDS, TB and Leprosy, controlling other diseases and provide ambulatory services, promote environmental sanitation, promote health education, monitor quality of water supplies, supervise and monitor the private sector services.

Currently, the District has one (1) general hospital, one (1) health centre four (IV), Seven (7) health centres III, and Twelve (12) health centres II. 8 health facilities are accredited to offer HAART services, all of which are implementing Differentiated services and delivery models. One hospital is Katakwi General Hospital, one health centre IV which is Toroma HC IV, 4 health centre III which include; Kapujan HCIII, Magoro HCIII, Aketa HCIII, Ngariam HCIII, and 2 private-not for profit (PNFP) facilities, which include St Ann Usuk HCIII and St Kevin Toroma HCIII.

The General hospital runs ART clinic Monday to Saturday every week whereas the health centers 3 and 4 all run ART clinics one day in a week. The cadres that run the ART clinics include; The medical clinical officers, registered midwives, enrolled midwives, enrolled nurses and counsellors. The above-mentioned cadres are helped by other lay workers including; the village health team members (VHTs), young adolescent peers (YAPs), Mentor mothers and expert clients who participate in patient care on scheduled clinic days but majorly constitute the community arm of care through follow-ups, drug delivery and community mobilization.

The Uganda health system is organized hierarchically according to the population they serve. The National Referral Hospital serves a population of 30,000,000, the Regional Referral Hospital 2,000,000, General Hospital 500,000, Health centre IV 70,000, Health Center III 20,000, Health Center II 5,000 and Health Center I (Village Health Team) 1000 people (Amelia et al, 2017).

### Study Population

The study population comprised of all clients diagnosed with HIV/AIDS and started on HAART by the year 2017 and are currently enrolled under any of the five differentiated service delivery approaches in any of the eight facilities offering HAART services in Katakwi District.

### Sample Size Calculation

The sample size for all the eight facilities was calculated from the formula below

$$\text{n}i=\frac{Z^{2}P\left(1-P\right)}{E}\text{n}i=\frac{{\left(2.58\right)}^{2}*0.5\left(1-0.5\right)}{\left(0.042\right)}\text{ n}i=943\text{ ART cards}.$$

To adjust sample size to our definitive population size of 4742 formula below was used.

$$\text{n}=\frac{ni}{1+\left(ni-1\right)/N}\text{ n}=\frac{943}{1+\left(943-1\right)/4742}\text{ n}=786\text{ ART cards}$$

so, the sample size is 786 ART cards

n*i* being sample size at infinite population

n being the adjusted sample size to the population of 4742 ART clients’ cards.

Z being the z-score at a given confidence level = 2.58 at 99% confidence level

p being the population proportion assumed to be 0.5%

E being margin of error which we used to be 4.2% or 0.042

N being the population size which is 4742 as the summation of the total number of clients in the 8 facilities that offer HAART services in the district.

The sample size for each health facility was obtained by probability proportionate to the size of the population size of each facility as shown in the Table 1 below.

#### Ratio

Was Obtained by dividing the cumulative number of clients on HAART in a particular facility by the total population (summation of cumulative number in all facilities).

#### Facility sample

Derived through the multiplication of the attained ratio by the sample size.

### Sampling Method

The purposive sampling method was used in the selection of Health facilities offering HAART services and rolled out DSD models.

A stratified random sampling method was used to pick samples from ART registers. A total number of clients active by December-2017 was obtained from ART registers which was divided by the sample size to get the sampling interval, files were arranged according to ART numbers but in their respective DSD models and samples picked according to the sampling interval until the sample size was reached for each health facility.

### Inclusion Criteria

ART cards of all HIV-positive clients who were enrolled on HAART in Katakwi district by the year 2017. This included children, adults, males and females.

### Exclusion Criteria

ART cards with incomplete data, ART cards of dead clients or transferred out and ART cards of clients enrolled on HAART after the year 2017.

### Data Collection Methods

Data collection was done by documentation review of HIV/ART care cards, viral load results reports, ART registers and quarterly reports of October - December 2021 and results entered into a predetermined data abstraction tool.

### Data Analysis

Raw data in our predetermined data abstraction tool was fed into excel sheets, analysed using the STATA software program in form of proportions of the numbers of the patients that have been retained and have a suppressed viral load and their determinants using logistic regression analysis.

## Results

### Baseline Characteristics

The total number of participants was 771 of which 42.7% were male and 57.3% were female with the mean age being 40 years. The other demographic characteristics (age, sex, marital status and level of the facility) are shown in Table 1. Clinical baseline characteristics (baseline CD4, baseline WHO clinical stage, initial HAART regimen and initial adherence) are shown in Table 2. Other clinical characteristics (DSD models, duration on HAART, co-morbidities, current adherence, current WHO clinical stage, retention and viral load outcome), see Table 3. The majority (86%) of the participants had been on ART for less than 6 years and the rest were above 6 years. Based on the MUAC 92.2% were well nourished, 1.4% had moderate acute malnutrition, whereas 6.4% had severe acute malnutrition.

### Retention rate

In this study, the clients reported as lost include the clients that were lost to follow-up, dead or self-transferred to other facilities. Clients who were active by 2017 and maintained an active follow-up throughout the follow-up period of 48 months were considered as retained. Lost to follow up are those clients who missed their appointments by 90 days and thereafter. The cumulative outcomes were; lost to follow-up 119(15.43%) and active follow-up was 651(84.57%) with a follow-up duration of 48 months. The corresponding retention rates at 95% CI of participants were 12 months (99.35%), 24 months (94.03%), 36 months (89.88%) and at 48months (84.57%). (Figs. 2 and 3)

### Viral load

**Figure 1:**
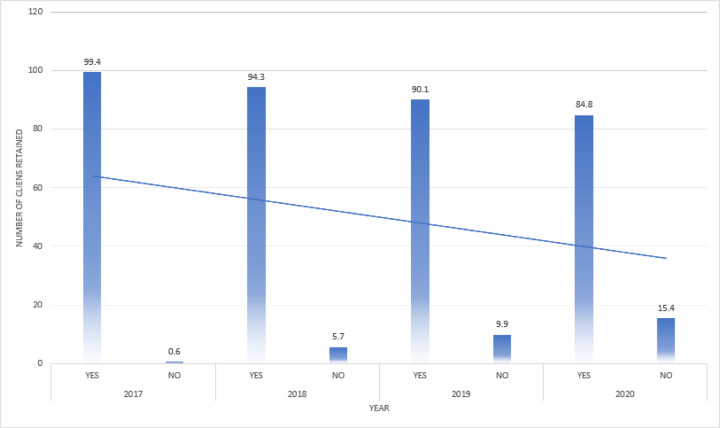
Number Of Clients on HAART Retained In Care from 2017–2020

In this study, a virally suppressed client was defined as a person whose HIV viral load was < 1000 copies/µl of blood. One major limitation encountered during data collection of viral load data was missing viral load results of up to 24.1% of clients which makes interpretation of viral data difficult. The cumulative outcomes of viral load were; suppressed 532(69%), non-suppressed 53(6.87%) and those without results 186(24.12%). The corresponding viral load suppression rates at 95% CI of participants were 12 months (57.3%, 11.9%, 33.8% for suppressed, non-suppressed and those without results respectively), 24 months (70.3%, 8.6%, 21.1% for suppressed, non-suppressed and those without results respectively), 36 months (70.3%, 8.3%, 20% for suppressed, non-suppressed and those without results respectively) and at 48 months (69%, 6.87%, 24.12% for suppressed, non-suppressed and those without results respectively. (Figs. 1 and 4)

#### Determinants of retention and viral load suppression.

Logistic regression analysis was performed to determine to what extent DSDM and other factors have contributed to viral suppression and retention among clients on HAART in Katakwi district. The determinants identified are shown in Table 4 as odds ratios of the outcomes (Table 5, table 6).

After adjustment in multivariate analysis, the significant determinants of retention were; age (OR 1.16, 95% CI: 0.89–1.52) being highest among 40–59 years and lowest in above 60 years, current adherence (OR 0.69, 95% CI: 0.27:1.74) being highest among clients with good adherence than poor adherence, current regimen (OR 4.53, 95% CI: 1.07–19.05) being highest among second regimen than first, DSD model (OR 0.87, 95% CI: 0.76–1.01) being highest in Community Drug Distribution Points and least in Facility Based Individual Management, and co-morbidities (OR 0.89, 95% CI: 0.70–1.12) with retention being highest among those with comorbidities.

Multivariate analysis showed that the significant determinants for viral load suppression were; age (OR 1.12, 95% CI:0.90–1.39) being highest among 40–59 and least in 0–19 age categories, facility level (OR 0.92, 95% CI:0.70–1.21) being highest at the general hospital and lowest at health center IV, sex (OR 1.02, 95% CI:0.73–1.43) being highest among females than males, marital status (OR 0.98, 95% CI:0.82–1.17) being highest amongst married and least among divorced, current adherence (OR 0.36, 95% CI:0.17–0.77) with the suppression higher with good adherence and lowest under fair, current regimen (OR 2.19, 95% CI:0.99–4.83) being highest among clients on first line and least on second line regimen, and type of DSD model (OR 0.96, 95% CI:0.87–1.07) being highest in Community Drug Distribution Points and lowest in facility based groups.

### Analysis of missing data

The missing data were treated and analysed as the rest of the data as shown in Table1, Table 2 and Table 3

Retention was highest among clients of 20–59 years of age, females, married and attending in a general hospital, whereas the percentage of viral load suppression was highest among 40–59 years of age, females, married and clients attending in General hospital (Table 2).

The retention and viral load suppression were highest amongst; Clients with initial CD4 201–400, Initial WHO clinical stage 1, good initial adherence and clients on first-line regimen (Table 3).

Adherence was obtained by getting number of pills divided by number of pills expected to be taken multiplied by 100 to get percentage adherence. Good adherence = 95% or more, fair adherence = 85–94% and poor = less than 85%.

For children, first line regimen children and adolescents include a combination of two nucleosides reverse transcriptase inhibitors plus one non-nucleosides reverse transcriptase inhibitors/a protease inhibitors (2NRTIs + NNRTIs or PIs) and second line is a combination two nucleosides reverse transcriptase inhibitors plus one integrase strand inhibitors or protease inhibitors (2 NRTI + 1PI/INSTI)

In adults, first line regimen is a combination of 2 nucleosides reverse transcriptase inhibitors plus one non-nucleoside reverse transcriptase inhibitor/a integrase strand inhibitor(2NRTIs + 1NNRTI/INSTI) and the second line having a combination of two nucleosides reverse transcriptase inhibitor and one protease inhibitor(2NRTIs + PI)

The majority of the clients were in FTDR. However, performance in both retention and Viral Load suppression were highest in CDDP. Retention and viral load suppression were highest among the following groups; 0–5 years of age, first-line regimen, good adherence and stage 1 WHO staging and PTB was the commonest co-morbidity (Table 4).

Retention of clients on HAART was highest in 2017 and reduced overtime with lowest in 2020.

Clients in CDDP had the highest percentage of Viral Load suppression whereas those in FBIM had the lowest.

The percentage of clients having their Viral load suppressed increased over time, being highest in 2019.

The percentage of clients with suppressed viral load was highest in CDDP and lowest in FBIM

## Discussion

Several studies conducted in rural settings of low and middle-income countries provide estimates of retention and viral load suppression and their determinants. However, most of these studies have not elucidated the impact differentiated service delivery approach has had on retention and viral load suppression in line with UNAIDS target of eradicating HIV epidemic by 2030.

In this study, we assessed for the sustained retention and viral load suppression and their determinants among clients attending HIV clinics in Eastern Uganda. We reviewed the ART data retrospectively of all clients that were active by 2017 and followed them through 2020.

It should be noted that clients in community models are pre-selected as stable clients before being assigned to the community-based models but can be changed to facility-based models should they become sick, develop opportunistic infections like meningitis, or become virally non-suppressing and returned when they become stable again and that irrespective of the approach, all clients were monitored for 3 years.

Generally, retention reduced steadily over time from 99.4% in 2017, 94.3% in 2018, 90.1% in 2019 and 84.8% in 2020 (Fig. 1). Albeit the decreasing trend overtime was expected and similar to most studies done in low- and middle-income countries, our results showed higher retention rates (Fig. 1). Similar studies in Jinja, Uganda in 2005–2009 showed retention after 48 months at 69% (13), in Tanzania being 83.9%, 64%, 53.5% by 12, 24 and 36 months respectively (14). In Malawi’s option B-plus program study being at 76.8%, 70.0% and 69.7% at 12, 24 and 36 months respectively (15), in another study on factors for attrition in Myanmar being at 86%, 82%, 80% and 77% at 12, 24, 36 and 48 months respectively (15). Other studies in Tigray, Ethiopia retention were 85.1% at 12 months and lastly in sub-Saharan Africa at 75% and 61.6 at 12 and 24 months respectively (16). The high retention in our study can be explained partly by the contribution of DSDM and the fact that most other studies were done before DSDM was rolled out as seen in one of the studies by Davison Kwarisima et al on Viral load and retention among adults and children using streamlined ART delivery in rural Uganda and Kenya where retention was at 92% (17).

**Figure 2:**
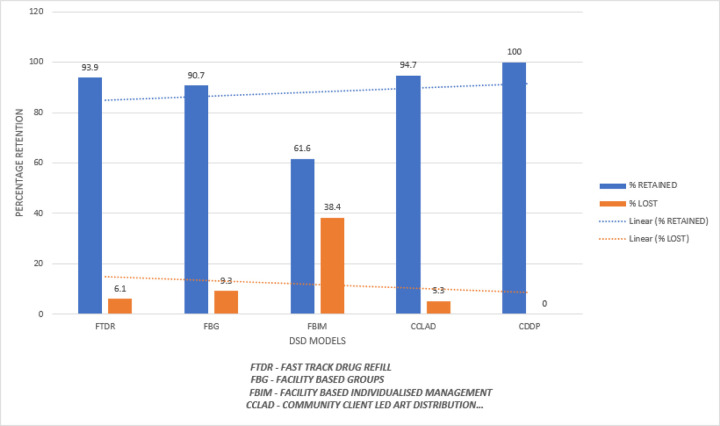
A bar graph showing percentage retention per DSD model over a period of 48 months (2017–2020)

Retention was highest among clients on HAART in the community based DSD models with 100% retention in CDDP, 94.7% in CCLAD compared to facility-based models in which FBIM performed the worst (61.6%, followed by FBG (90.7%) and FTDR (93.9%) (Fig. 2). This is in line with a similar study by Kagimu et al on overcoming barriers to access HIV services among female sex workers in a CCLAD in TASO Entebbe where retention improved from 65–100% in one year and another similar study on streamlined ART(18). This can be explained by good adherence associated with community models due to reduced clinic visits, transport costs and enhanced psycho-social support as shown by Anna Grimsrud et al in a study of the evidence for scale up differentiated care research agenda (19). Asseta et al in a study in Ethiopia who noted community based care as one of the retention promoting activities for clients on HAART(20) and by L. Prust et al in 2016 in Malawi who made the same observation(21). The higher retention rates in FTDR than in the rest of the facility models could be explained by reduced waiting time as noted by Anna Grimsrud et al in evidence for scaling up of differentiated care studies (19) and provision of thorough checks as noted by Vicent Adjetey et al in a study of people living with HIV accessing tertiary institutions in Ghana (6).

Other predictors of retention according to the multivariate analysis included; being 40–59 years of age (OR 1.16, 95% CI:0.89–1.52), having good current adherence (OR 0.69, 95% CI:0.27:1.74), being on current first-line regimen (OR 4.53,95% CI:1.07–19.05) and having no co-morbidities (OR 0.89, 95% CI:0.70–1.12), albeit pulmonary tuberculosis (PTB) had the highest retention (2.24%) among the co-morbidities (Table 5). This is in line with a study on retention in Jinja, Uganda by Stephen Okoboi who highlighted female gender as a predictor of better retention (13) and being on TB treatment by Haas et al in Myanmar (15).

**Figure 3:**
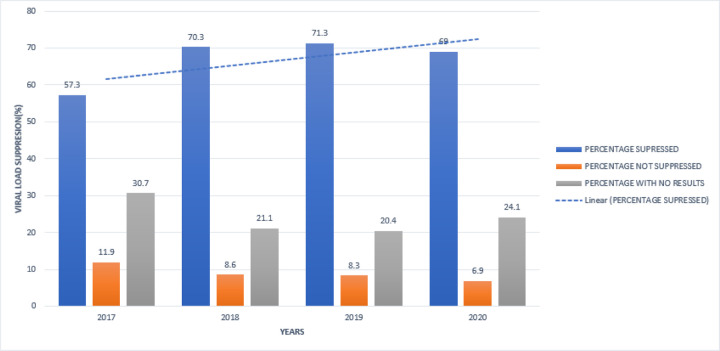
Viral Suppression Of Art Clients from 2017–2020

Viral load suppression in general improved over time from 57.3% in 2017, 70.3% in 2018, 71.3% in 2019 and 69% in 2020 (Fig. 3). This was partly due to the introduction of differentiated service delivery models which tailored care to the best individual needs. This was contrary to one of the studies by Jonathan Colosanti et al on continuous retention and viral load suppression in the HIV care continuum who noted 64%, 48% and 39% at 12, 24 and 36 months respectively (4) but similar to the result noted by Bijal Shah et al of 63% at 12 months (22). Our results, however, were very low compared to one of the studies that were done by Daltons Kwarisima et al of 93% at 12 months (23).

**Figure 4:**
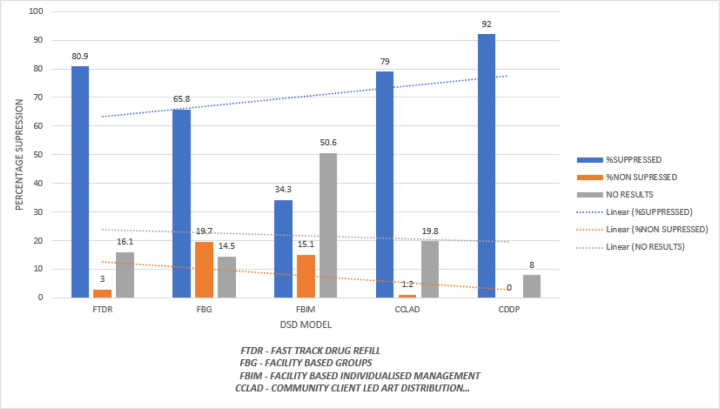
Suppression per DSD model over a period of 48 month

Viral load suppression was highest amongst the community based DSD models in which CDDP performed the best (92%) followed by CCLAD (79%) compared to the facility based DSD models in which FBIM performed the worst (34.3%) with the majority of clients having no results, FBG (65%) with FTDR being exceptional (80.9%) (Fig. 4). This is in line with the study by Kagimu et al on overcoming barriers to access of HIV/AIDS services among female sex workers through DSD in CCLAD at TASO Entebbe-Uganda who noted an increase in viral load suppression from 80–100% in 12 months (18). This can be explained partly by the fact that clients on HAART in the community based model easily access care at convenience with fewer clinic visits, spending less on transport costs and good adherence associated with enhanced psycho-social support as opposed to facility based model reported, L. Prust et al in a study of patients and health workers’ experience of DSD model for stable patients in Malawi (21).

Other predictors of viral suppression from the multivariate analysis include; being 40–59 years (OR 1.12, 95% CI:0.90–1.39), receiving care from the general hospital (OR 0.92, 95% CI:0.70–1.21), being married (OR 0.98, 95% CI:0.82–1.17), having good current adherence (OR 0.36, 95% CI:0.17–0.77), being on first line of the current regimen (OR 2.19, 95% CI:0.99–4.83) and being a female (OR 1.02, 95% CI:0.73–1.43) (Table 6). It was also noted by Bijal Chah that first line HAART regimens and good adherence are some of the predictors of viral load suppression (22).

### Limitations

One major limitation during data collection was documentation gap where most files had DSD model approaches missing especially in the initial years of 2017 to 2018. This could be attributed to the fact that DSDM had just been enrolled and health workers had not appreciated it well and secondly the Health Management information System (tools) tools had not been customised to DSDM making it hard to document information concerning DSDM.

It was also noted that health workers still had knowledge gap in re-assessing and re-categorising clients at each visit into the suitable DSD model evidenced having non-suppressing clients in CCLAD which are meant for facility models and having so many suppressing clients in FTDR and FBIM and yet qualify for community model.

Incomplete documentation of information in primary data tools like ART registers and missing data for some periods in registers, for example for a given week or month.

A high percentage of the clients were missing Viral load results (24.12%) making interpretation of the results difficult.

## Conclusions

Both facility and community based DSD models have led to improved and hence sustained retention and viral load suppression through mitigation of barriers to effective HIV/AIDS care of clients on highly active anti-retrovirus therapy (HAART) including the needs of sub-populations, albeit clients that started from community based DSD models have shown to a chieve better sustained outcomes than facility based DSD models. Viral load suppression has remained below the UNAIDs target of 90% by 2020 and 95% by 2030 although it improved over time.

### Recommendations

More resources should be allocated to facilitate community-based models, especially CDDP, which were found to have very few clients and yet evidence shows that they achieve better retention and viral suppression.

The DSD committee should be activated to start a QI project to ensure that all clients due for viral load are done, followed up and properly documented to reduce cases of no results. On top of this, the non-suppressing clients should be optimized to regimens by doing drug sensitivity testing for those not suppressing.

There is a need to bring the community-based organizations rendering psycho-social support to clients on board to improve compliance as this has been shown to yield good results.

The knowledge gaps identified can be addressed by continuous support supervision, onsite mentorships and employment of quality improvement measures.

The identified documentation gaps can be reduced by provision and use of data tools customised for DSD models on top of continuous support supervision to encourage health workers complete the documentation in registers.

### Generalizability

Our findings can be generalized to similar settings in Uganda and other developing countries where these models have been rolled out.

## Figures and Tables

**Table 1:** Distribution of the Sample Size among Selected Health Facilities in Katakwi District.

Health Facility	Ratio	Facility Sample	Number of Clients Active on HAART by 2017
Katakwi General Hospital	0.50	393	2431
Ngariam HC III	0.07	55	295
Magoro HC III	0.11	88	547
Aketa HC III	0.09	71	421
Toroma HC IV	0.10	77	464
Usuk HC III	0.06	47	264
Kapujan HC III	0.05	39	237
St Kevin Toroma HC III	0.02	16	83
**Total**	**1**	**786**	**4742**

**Table 2:** Demographic characteristics of study participants

Variable		Retention			Viral load suppression			
		Retained(n/%)	Lost (n/%)	(N/%)	Suppressed (n/%)	Non-Suppressed (n/%)	Missing	(N/%)
Age	0–19	78(11.62)	5(5.0)	**83(10.77)**	53(9.96)	18(33.96)	12(6.45)	**83(10.77)**
	20–39	244(36.36)	57(57.0)	**301(39.04)**	189(35.53)	15(28.30)	97(52.15)	**301(39.04)**
	40–59	277(41.28)	27(27.0)	**304(39.43)**	226(42.48)	18(33.96)	60(32.260	**304(39.43)**
	60-above 79	72(10.73)	41.28(11.0)	**83(10.77)**	64(12.03)	2(3.77)	17(9.14)	**83(10.77)**
	**Total**	**671(87)**	**100(13)**	**771(100)**	**532(69)**	**53(6.87)**	**186(24.13)**	**771(100)**
Sex	male	295(43.96)	34(34)	**329(42.67)**	216(40.6)	35(66.04)	78(41.94)	**329(42.67)**
	female	376(56.04)	66(66)	**442(57.33)**	316(59.4)	18(33.960	108(58.06)	**442(57.33)**
	**Total**	**671(87)**	**100(13)**	**771(100)**	**532(69)**	**53(6.87)**	**186(24.13)**	**771(100)**
Marital status	single	136(20.270	17(17)	**153(19.84)**	96(18.05)	22(41.51)	35(18.82)	**153(19.84)**
	married	426(63.49)	59(59)	**485(62.99)**	346(65.04)	27(50.94)	112(60.22)	**485(62.91)**
	widow	28(4.17)	7(7)	**35(4.540**	26(4.89)	0(0)	9(4.54)	**35(4.54)**
	divorced	8(1.19)	2(2)	**10(1.3)**	7(1.37)	0(0)	3(1.61)	**10(1.3)**
	Missing results	73(10.88)	15(15)	**88(11.41)**	57(10.71)	4(7.55)	27(14.52)	**88(11.41)**
	**Total**	**671(87)**	**100(13)**	**771(100)**	**532(69)**	**53(6.87)**	**186(24.13)**	**771(100)**
facility level	HC III	276(41.13)	40(40)	**316(40.99)**	210(39.47)	32(60.38)	74(39.788)	**316(40.99)**
	HC IV	36(5.37)	11(11)	**47(6.10)**	30(5.64)	1(1.89)	16(8.6)	**47(6.1)**
	General Hospital	359(53.50)	49(49)	**408(52.92)**	292(54.89)	20(37.74)	96(51.61)	**408(52.92)**
	**Total**	**671(87)**	**100(13)**	**771(100)**	**532(69)**	**53(6.87)**	**186(24.13)**	**771(100)**

**Table 3;:** Baseline characteristics of the study participants

variable		Retention			Viral load suppression			
		Retained	Lost	(N/%)	Suppressed	Non - suppressed	missing	(N/%)
Initial CD4	1–200	100(14.90)	13(13)	**113(14.66)**	82(15.41)	8(15.09)	23(12.37)	**113(14.66)**
	201–400	139(21.02)	29(29)	**168(21.79)**	120(22.56)	6(11.32)	42(22.58)	**168(21.79)**
	401–600	95(14.16)	11(110)	**106(13.75)**	79(14.85)	8(15.09)	19(10.22)	**106(13.75)**
	601–800	62(9.24)	4(4)	**66(8.56)**	49(9.21)	2(3.77)	15(88.06)	**66(8.56)**
	Above 800	59(8.79)	11(11)	**70(9.08)**	48(9.02)	3(5.66)	19(10.22)	**70(9.08)**
	missing	216(32.19)	32(32)	**248(32.17)**	154(28.95)	26(49.06)	68(36.56)	**248(32.17)**
	**Total**	**671(87)**	**100(13)**	**771(100)**	**532(69)**	**53(6.87)**	**186(24.13)**	**771(100)**
Initial WHO stage	1	402(59.91)	56(56)	**458(59.40)**	323(60.71)	30(56.60)	105(56.45)	**458(59.40)**
	2	193(28.76)	31(31.0)	**224(29.05)**	148(27.82)	18(33.96)	58(31.18)	**224(29.05)**
	3	73(10.88)	11(11.0)	**84(10.89)**	58(10.90)	5(9.43)	21(11.29)	**84(10.89)**
	4	3(0.45)	2(2.0)	**5(0.65)**	3(0.56)	0(0)	2(1.08)	**5(0.65)**
	**Total**	**671(87)**	**100(13)**	**771(100)**	**532(69)**	**53(6.87)**	**186(24.13)**	**771(100)**
Initial regimen	first	658(98.31)	98(98.0)	**756(9.05)**	526(98.85)	48(90.57)	183(98.39)	**576(98.05)**
	second	12(1.79)	2(2)	**14(1.82)**	6(1.13)	5(9.43)	3(1.61)	**14(1.82)**
	**Total**	**671(87)**	**100(13)**	**771(100)**	**532(69)**	**53(6.87)**	**186(24.13)**	**771(100)**
Initial adherence	good	641(95.53)	92(92.0)	**733(955.07)**	513(96.43)	49(92.45)	171(91.94)	**733(98.07)**
	Fair	10(1.49)	1(1.0)	**11(1.43)**	7(1.32)	1(1.89)	3(1.61)	**11(1.43)**
	Poor	20(2.98)	7(7.0)	**27(3.5)**	12(2.26)	3(5.66)	12(6.45)	**27(3.5)**
	**Total**	**671(87)**	**100(13)**	**771(100)**	**532(69)**	**53(6.87)**	**186(24.13)**	**771(100)**

**Table 4:** Other clinical characteristics of the study participants.

variable		retention			Viral load suppression			
DSD models		Retained	Lost	(N/%)	Suppressed	Non suppressed	missing	(N/%)
	FTDR	306(45.60)	20(20)	326(42.28)	264(49.62)	10(18.87)	52(27.96)	**326(42.28)**
	FBG	69(10.28)	7(7)	76(9.86)	50(9.4)	15(28.30)	11(5.91)	**76(9.86)**
	FBIM	108(16.01)	64(64)	172(22.31)	59(11.09)	26(49.06)	87(46.77)	**172(22.31)**
	CCLAD	163(24.29)	9(9)	172(22.31)	136(25.56)	2(3.77)	34(18.28)	**172(22.31)**
	CDDP	25(3.73)	0(0)	25(3.24)	23(4.32)	0(0)	2(1.08)	**25(3.24)**
	**Total**	**671(87)**	**100(13)**	**771(100)**	**532(69)**	**53(6.87)**	**186(24.13)**	**771(100)**
Duration on HAART (years)	0–5	524(78.09)	85(85.0)	609(78.99)	407(76.50)	44(83.02)	158(84.95)	**608(78.99)**
	6–10	134(19.97)	14(14)	148(19.2)	114(21.43)	8(15.09)	26(13.98)	**148(19.2)**
	11–15	11(1.64)	1(1)	12(1.56)	9(1.69)	1(1.89)	2(1.08)	**12(1.56)**
	16 and above	2(0.3)	0(0)	2(0.26)	2(0.38)	0(0)	0(0)	**2(0.26)**
	**Total**	**671(87)**	**100(13)**	**771(100)**	**532(69)**	**53(6.87)**	**186(24.13)**	**771(100)**
comorbidities	PTB	15(2.24)	4(4)	19(2.46)	10(1.88)	5(9.43)	4(2.15)	**19(246)**
	PJP	1(0.15)	0(0)	1(0.13)	1(0.19)	0(0)	0(0)	**1(0.13)**
	HTN & DM	1(0.15)	1(1)	2(0.26)	1(0.19)	0(0)	1(0.54)	**2(0.26)**
	malnutrition	1(0.15)	2(2)	3(0.39)	1(0.19)	0(0)	2(1.08)	**3(0.39)**
	Cry meningitis	1(0.15)	1(1)	2(0.26)	0(0)	0(0)	2(1.08)	**2(0.26)**
	fungal infection	3(0.45)	1(1)	4(0.52)	3(0.56)	0(0)	1(0.54)	**4(0.52)**
	other	21(3.13)	4(4)	25(3.24)	16(3.01)	1(1.89)	8(4.30)	**25(3.24)**
	none	628(93.59)	87(87)	715(92.74)	500(93.98)	47(88.68)	168(90.32)	**715(92.74)**
	**Total**	**671(87)**	**100(13)**	**771(100)**	**532(69)**	**53(6.87)**	**186(24.13)**	**771(100)**
Current regimen	first	619(92.25)	98(98)	717(93.0)	503(94.55)	36(67.92)	178(95.7)	**717(93.0)**
	second	52(7.75)	2(2)	54(7.0)	29(5.45)	17(32.08)	8(4.30)	**54(7.0)**
	**Total**	**671(87)**	**100(13)**	**771(100)**	**532(69)**	**53(6.87)**	**186(24.13)**	**771(100)**
Current adherence	good	646(96.27)	89(89)	735(95.33)	521(97.93)	48(90.57)	166(89.25)	**735(95.33)**
	fair	8(1.19)	4(4)	12(1.56)	5(0.94)	2(3.77)	5(2.69)	**12(1.56)**
	poor	17(2.53)	7(7)	24(3.11)	6(1.13)	3(5.66)	15(8.06)	**24(3.11)**
	**Total**	**671(87)**	**100(13)**	**771(100)**	**532(69)**	**53(6.87)**	**186(24.13)**	**771(100)**
WHO clinical stage	1	596(88.96)	85(85.86)	681(88.56)	487(91.71)	38(71.70)	156(84.32)	**681(88.56)**
	2	68(10.15)	12(12.12)	80(10.40)	40(7.53)	13(24.53)	27(14.59)	**80(1040)**
	3	6(0.90)	2(2.02)	8(1.04)	4(0.75)	2(3.77)	2(1.08)	**8(1.04)**
	**Total**	**671(87)**	**100(13)**	771(100)	**532(69)**	**53(6.87)**	**186(24.13)**	**771(100)**

**Table 5:** Determinants of retention to care

retention	Odds Ratio	P-value	[95% Conf. Interval]
Age	1.163117	0.001	0.888887–1.521949
Current adherence	0.6868554	0.005	0.27021–1.74594
Current regimen	4.528328	0.036	1.076053–19.05645
Type of DSD model	0.875006	0.001	0.758093–1.00995
Co-morbidities	0.8875778	0.037	0.70142–1.123142

**TABLE 6:** Determinants of viral load suppression.

Viral load suppression	Odds Ratio	P-value	[95% Conf. Interval]
Age	1.120251	0.001	0.903982–1.388262
Facility level	0.9221916	0.020	0.700178–1.214602
Sex	1.022738	0.002	0.729055–1.434725
Marital status	0.9788787	0.007	0.817738–1.171773
Current adherence	0.3587538	0.001	0.166869–0.771289
Current regimen	2.189893	0.001	0.992561–4.831575
Type of DSD model	0.9656563	0.001	0.867439–1.074995

## Abbreviations

UNAIDS: United Nations Program on HIV/AIDS
DSD: Differentiated Service Delivery
ART: Anti-Retroviral Therapy
CDDP: Community Drug Distribution Points
CCLAD: Community Client Led ART Delivery
FBIM: Facility Based Individualised Management
FBG: Facility Based Groups
FTDR: Fast Track Drug Refills
HIV: Human Immunodeficiency Virus
AIDS: Acquired Immune Deficiency Syndrome
US: United States
SPEEDI: Standardized Paediatrics Expedited Encounters for Drugs
CAD: Community ART Distribution
TB: Tuberculosis
HC: Health Centre
WHO: World Health Organization
MUAC: Mid Upper Arm Circumference
CD4: Cluster of Differentiation 4
CI: Confidence interval
OR: Odds Ratio
PTB: Pulmonary Tuberculosis
TASO: The AIDS Support Organisation
QI: Quality Improvement
MRRHREC: Mbale Regional Referral Research and Ethics Committee
